# Membranous Nephropathy in a Patient with Human Immunodeficiency Virus Shortly After Initiation of HAART with Atripla

**DOI:** 10.7759/cureus.3932

**Published:** 2019-01-21

**Authors:** Muhammad Shabbir Rawala, James Wright, Judy King, David Howell, Shelda Martin

**Affiliations:** 1 Internal Medicine, Charleston Area Medical Center, Charleston, USA; 2 Neurosurgery, University Hospitals Cleveland Medical Center, Cleveland, USA; 3 Pathology, Louisiana State University, Shreveport, USA; 4 Internal Medicine, Duke University, Raleigh, USA

**Keywords:** nephritis, tenofovir, atripla, hiv

## Abstract

A human immunodeficiency virus (HIV) infection has long been associated with kidney disease. The pathogenesis of renal complications may be directly related to the presence of HIV viral particles or may occur secondary to an immune response against the virus. A number of HIV medications have been associated with the development of acute and chronic kidney disease. It has been estimated that approximately 60 percent of patients suffering from HIV/acquired immunodeficiency syndrome (AIDS) will, at some point, manifest clinically significant renal sequelae.The most common kidney disease affecting HIV patients is HIV-associated nephropathy (HIVAN) or focal segmental glomerulonephritis (FSGS). A very small subset of patients suffering from HIV/AIDS does go on to develop membranous glomerulonephritis. We present a case of a 55-year old Caucasian male who presented to the hospital after two weeks of weakness and falling when attempting to stand. The patient had a history of HIV, diagnosed in 1996. The latest cluster differentiation 4 (CD4) count was 245 cells/uL and the HIV-ribonucleic acid (RNA) viral load was reported as less than 75 copies/ml. The physical exam at presentation was insignificant. The laboratory examination revealed elevated creatinine. Potential nephrotoxic home medications, including Atripla and lisinopril, were held. After a brief inpatient stay, he was discharged but was ultimately readmitted for worsening renal function and nephrotic syndrome was diagnosed. Renal biopsy was performed, and membranous glomerulonephritis was confirmed. To this point, there are no associated cases reported of membranous glomerulonephritis after initiation of therapy with Atripla.

We present a case of a rare etiology of membranous nephropathy in an HIV patient. Physicians should be judicious in detecting the etiology of renal dysfunction in HIV patients.

## Introduction

A human immunodeficiency virus (HIV) infection has long been associated with kidney disease [1-5].Kidney disease may be directly related to the presence of HIV particles or occur secondary to an immune response against these particles [2].A number of HIV medications have also been shown to cause renal abnormalities. It has been estimated that approximately 60% of patients with AIDS will manifest a clinically significant kidney disease [4].The most common kidney diseases affecting HIV patients include HIV-associated nephropathy (HIVAN) or focal segmental glomerulosclerosis (FSGS) [1,4,6].Membranous glomerulonephritis (MN) is only rarely seen concomitantly with an HIV infection; however, it is a common cause of nephrotic syndrome in Caucasians [2,5,7]. MN is characterized microscopically by glomerular basement membrane (GBM) thickening without hypercellularity and subepithelial immune complex deposits [8].

## Case presentation

**Figure 1:**
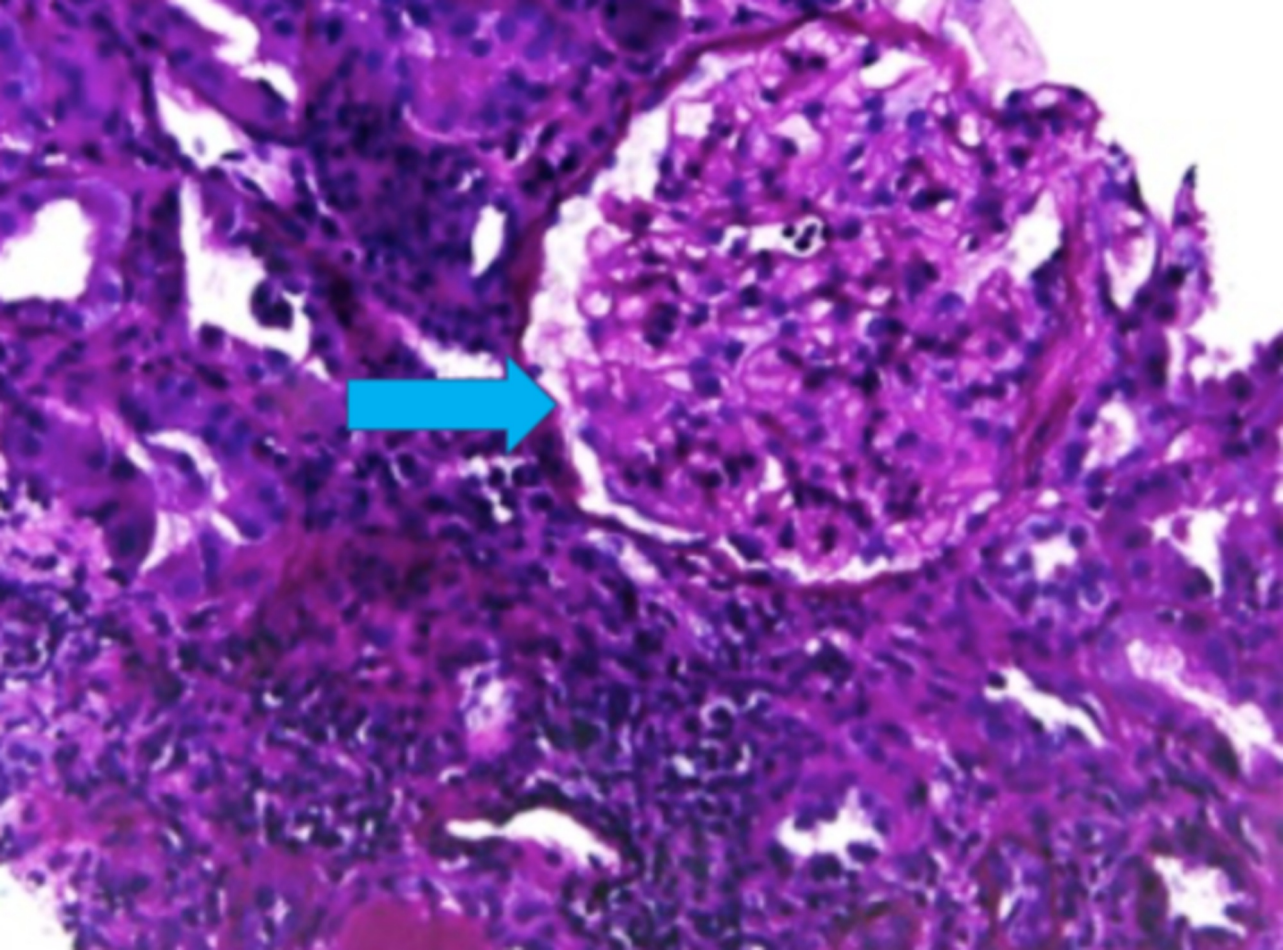
PAS stain showing thickened glomerular capillaries (arrow) signifying membranous nephropathy Periodic acid–Schiff (PAS)

**Figure 2:**
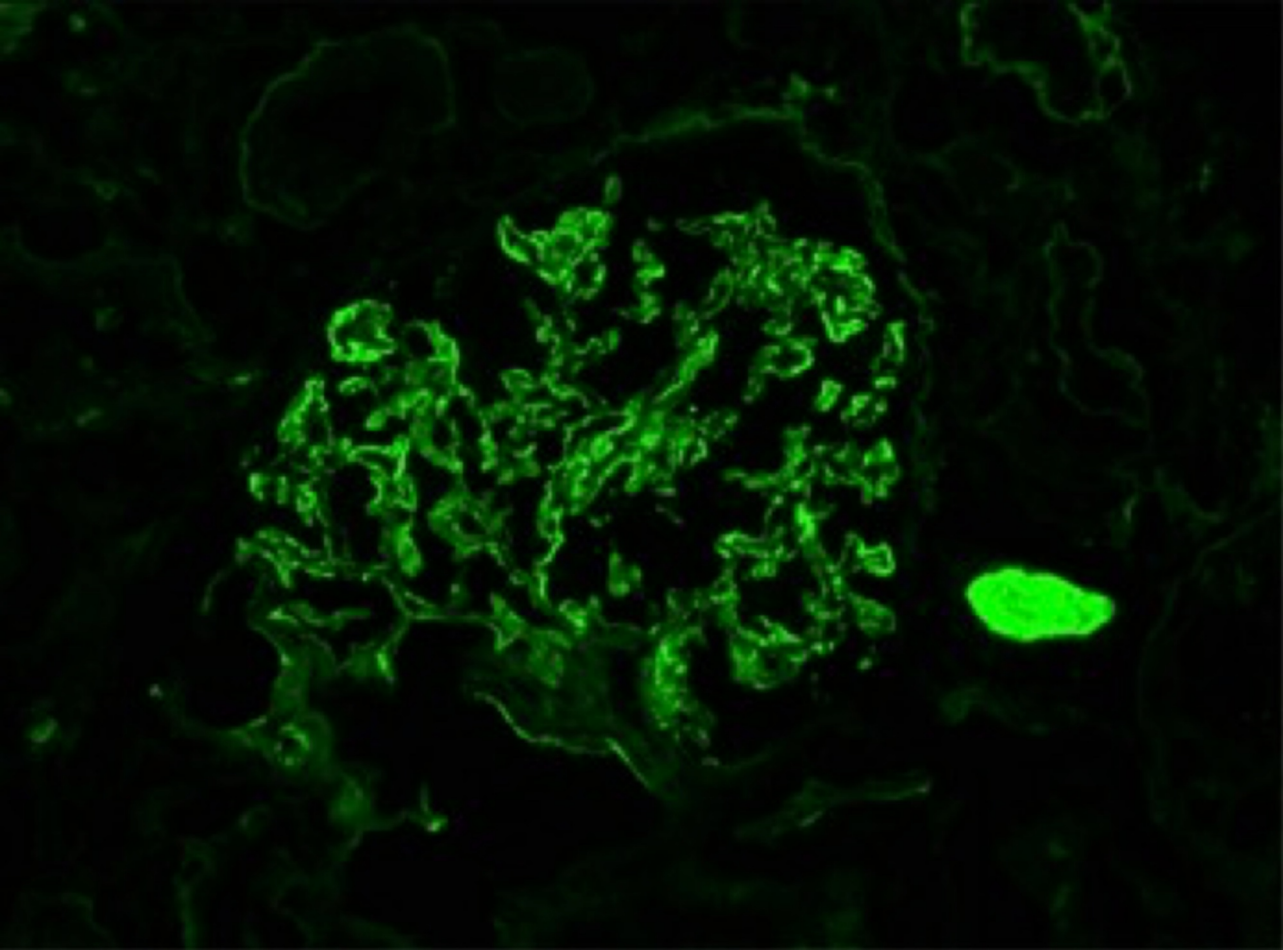
IgG immunofluorescence indicating IgG deposits in renal glomeruli Immunoglobulin G (IgG)

**Figure 3:**
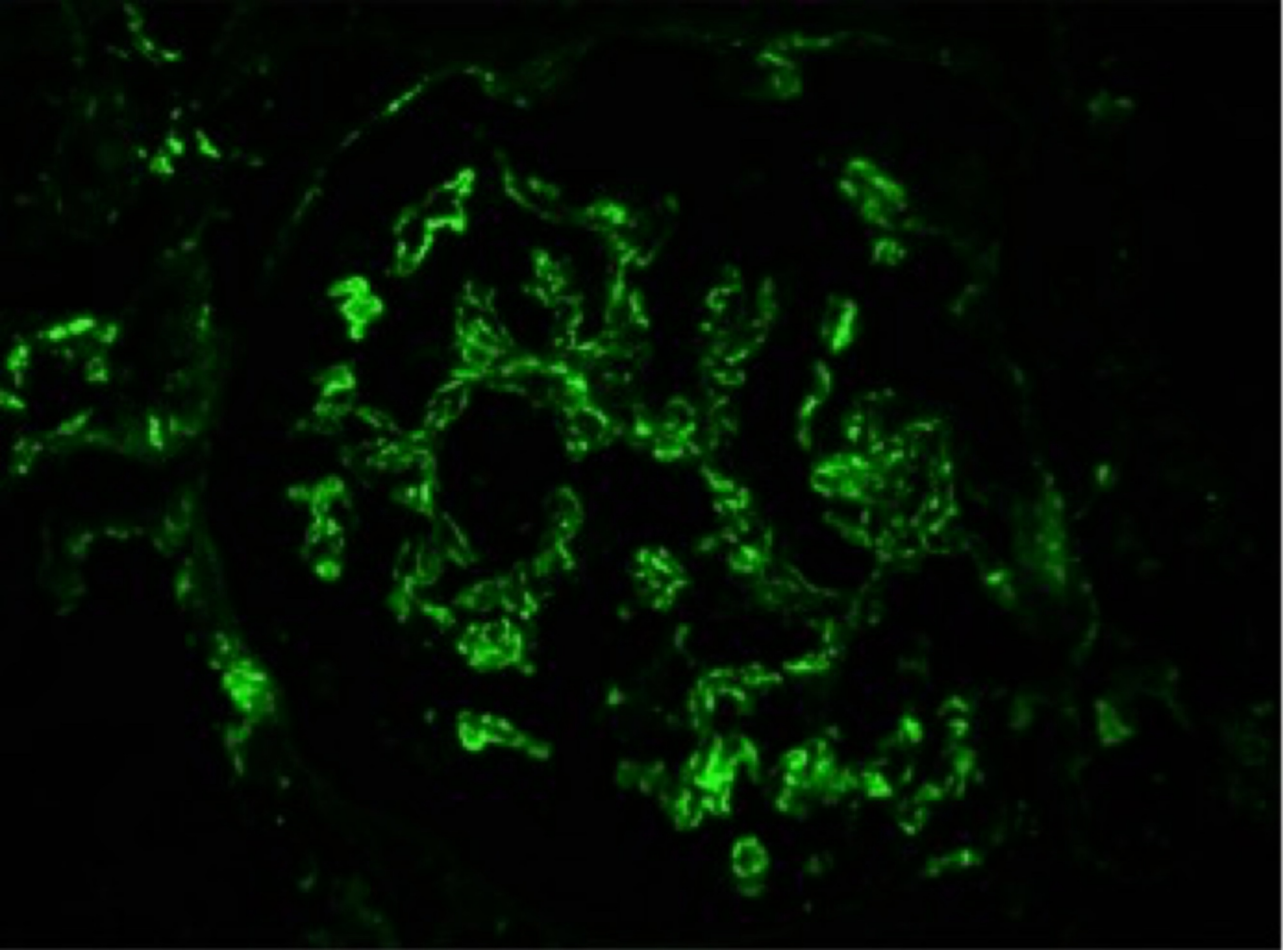
Complement C3 immunofluorescence indicating C3 deposits in renal glomeruli

**Figure 4:**
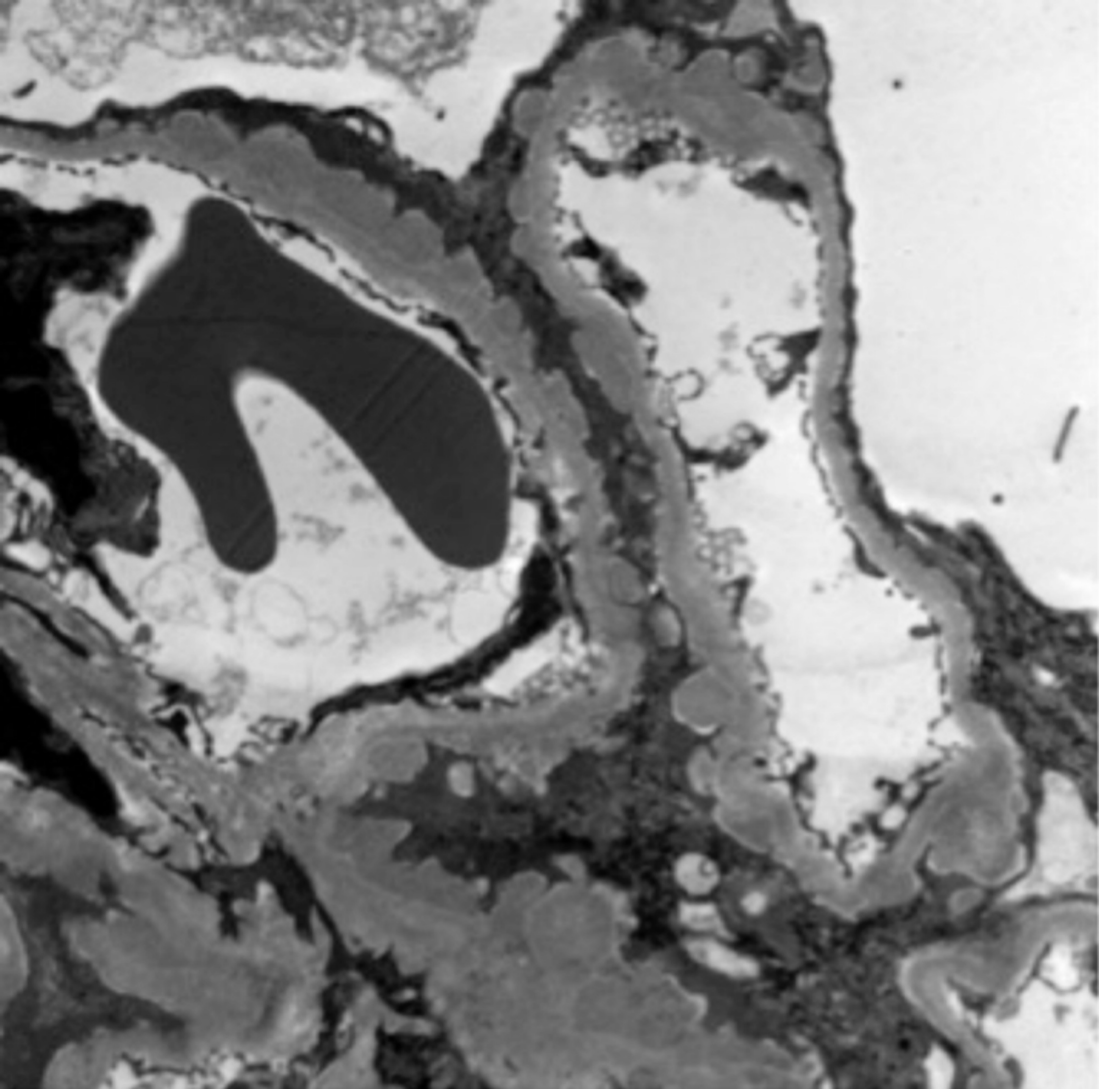
Electron microscopy indicating the presence of sub-epithelial deposits

A 55-year-old Caucasian male with a history of HIV diagnosed in 1996, whose cluster differentiation 4 (CD4) count was 245 cells per microliter and HIV-ribonucleic acid (RNA) was less than 75 copies per milliliter, presented to the emergency department with the primary complaint of two weeks of weakness and multiple falls. The patient’s comorbid conditions were significant for hypertension (HTN), hyperlipidemia (HLD), anemia, hypogonadism, pancreatitis, peripheral neuropathy, and chronic pain (managed with opiate medication and not with any nonsteroidal anti-inflammatory drugs (NSAIDs)). He denied any other complaints. The physical examination was significant for facial ecchymoses. The laboratory examination yielded an elevated creatinine at 2.8 mg/dL. The patient had no history of previous kidney disease and had been followed regularly by his primary care physician. Potential nephrotoxic home medications, including Atripla and lisinopril, were stopped at the time of presentation and the patient underwent full workup for new acute kidney injury (AKI). Of note, the patient had been on lisinopril for a number of years; however, he had begun therapy with Atripla approximately 170 days prior to this presentation. The initial workup yielded no results. The patient was discharged home but returned multiple times with sequelae of worsening creatinine and ultimately developed the nephrotic syndrome. Further workup of Fanconi syndrome also proved negative. Ultimately, a renal biopsy was performed, which helped in establishing the patient’s diagnosis as MN (Figures 1-4). The patient was managed conservatively with steroids only, to which his renal function responded minimally but stabilized. The patient was further followed up as an outpatient with a nephrologist.

## Discussion

Mostly, HIV patients develop focal segmental glomerulosclerosis (FSGS) as a cause of nephrotic syndrome. Membranous glomerulonephritis (MGN) is very infrequently seen in patients with HIV. MN can be idiopathic or secondary to any other etiology, however, the idiopathic type is more common [8]. Secondary causes of MN has been associated with drugs, malignancies, autoimmune diseases, and chronic infections. The management of secondary MN focuses on the treatment of the underlying cause.

The most common cause of nephrotic syndrome in adult Caucasians is membranous glomerulonephritis. The disease is characterized by subepithelial immune complex deposits of immunoglobulin G (IgG) and complement (C3) in the glomerulus, along with a thickening of the capillary walls visualized by light microscopy [7]. This thickening is a result of glomerular inflammation via the membrane attack complex C5b-9, which ultimately leads to a loss of protein and nephrotic range proteinuria (>3.5 g/24hr). The goals of management primarily in HIV positive patients typically target the suppression of viral load and the reduction of the advancement of renal disease with medications such as angiotensin-converting enzyme (ACE) inhibitors for symptomatic therapy and alternating cytotoxic agents and corticosteroids [1-2].

Patients with HIV are at a four-fold higher risk for developing chronic kidney disease (CKD) or end-stage renal disease (ESRD) due not only to the inherently increased risk from HIV but also due to several frequently seen co-morbidities, including co-infection with hepatitis C virus (HCV), HTN, diabetes mellitus, age, low CD4 count, and high HIV viral load [3-4]. Studies have shown HIV-associated nephropathy (HIVAN) to be a result of HIV viral replication in renal epithelial cells, which corresponds with findings that highly active antiretroviral therapy (HAART) can improve HIVAN [3]. However, studies have shown that in patients with renal disease other than HIVAN, antiretroviral therapy did not result in an improvement in renal function [5].

A number of medications commonly used for HAART therapy may attribute to renal dysfunction and disease. The patient presented herein was taking the combination drug tenofovir, efavirenz, emtricitabine (Atripla). Of these three medications, only tenofovir has been previously associated with nephrotoxicity [6]. The most common presentation of tenofovir-associated renal toxicity is acute renal failure, nephrogenic diabetes insipidus, and Fanconi syndrome, characterized by renal tubular acidosis, glycosuria with normoglycemia, aminoaciduria, hypophosphatemia, hypouricemia, and tubular proteinemia [4,9-10]. Existing data suggest that the mechanism of damage is due to decreased molecular transportation into the mitochondria of the renal proximal tubule cells [10].

Upon a review of the literature, no causal association between membranous nephropathy and HIV has been identified, although a few case reports have been published in the past, which indicate that some association may exist [5]. However, no known association exists between tenofovir, or the combination drug Atripla, and the development of membranous glomerulonephritis.

## Conclusions

This case is significant given the widespread use of tenofovir for the treatment of HIV. Further studies are necessary on the development of membranous glomerulonephritis in patients with HIV and to elucidate any possible role that tenofovir, or the combination drug Atripla, may play in the development of this disease. In addition, individuals being treated with single agents, such as efavirenz or emtricitabine, may also need to be followed up for development of MGN.
